# Serological investigation of asymptomatic cases of SARS-CoV-2 infection reveals weak and declining antibody responses

**DOI:** 10.1080/22221751.2021.1919032

**Published:** 2021-05-18

**Authors:** Yong Yang, Xi Wang, Rong-Hui Du, Wei Zhang, Hao-Rui Si, Yan Zhu, Xu-Rui Shen, Qian Li, Bei Li, Dong Men, Ya-na Zhou, Hui Wang, Xiao-lin Tong, Xian-en Zhang, Zheng-Li Shi, Peng Zhou

**Affiliations:** a CAS Key Laboratory of Special Pathogens & State Key Laboratory of Virology, Wuhan Institute of Virology, Center for Biosafety Mega-Science, Chinese Academy of Sciences, Wuhan, People’s Republic of China; b University of Chinese Academy of Sciences, Beijing, People’s Republic of China; c Wuhan pulmonary hospital, Wuhan, People’s Republic of China; d Hubei provincial hospital of TCM, Wuhan, People’s Republic of China; e Wuhan Municipal Health Commission, Wuchang branch, Wuhan, People’s Republic of China; f Department of Endocrinology, Guang'anmen Hospital, China Academy of Chinese Medical Sciences, Beijing, 100053, People’s Republic of China; g National Laboratory of Biomacromolecules, CAS Center for Excellence in Biomacromolecules, Institute of Biophysics, Chinese Academy of Sciences, Beijing 100101, People’s Republic of China; h Joint Laboratory of Infectious Diseases and Health, Wuhan Institute of Virology & Wuhan Jinyintan Hospital, Wuhan, People’s Republic of China

**Keywords:** SARS-CoV-2, COVID-19, asymptomatic, serology, weak antibody response

## Abstract

Without an effective vaccine against SARS-CoV-2, the build-up of herd immunity through natural infection has been suggested as a means to control COVID-19. Although population immunity is typically estimated by the serological investigation of recovered patients, humoral immunity in asymptomatic subjects has not been well studied, although they represent a large proportion of all SARS-CoV-2 infection cases. In this study, we conducted a serosurvey of asymptomatic infections among food workers and performed serological and cellular response analyses of asymptomatic subjects in Wuhan, the original epicenter of the COVID-19 outbreak. Our data showed that up to 5.91% of the food workers carried SARS-CoV-2 IgG antibodies asymptomatically; however, in 90.4% of them, the antibody level declined over a 2-week period. IgM and IgG antibodies, including neutralizing antibodies, were significantly lower in asymptomatic subjects than in recovered symptomatic patients with similar disease courses. Furthermore, the asymptomatic subjects showed lymphopenia and a prominent decrease in the B-cell population, as well as a low frequency of antibody-secreting cells and a low cytokine response. These factors probably contributed to the low and unsustained antibody levels in asymptomatic subjects. Our results show that asymptomatic subjects are likely to be vulnerable to SARS-CoV-2 reinfection, and neither the proportion of population immunity nor the breadth of immune responses is sufficient for herd immunity.

## Introduction

Since the first outbreak in Wuhan, China, COVID-19, which is caused by SARS-CoV-2, has spread and caused a serious public health crisis globally. To date (April 13, 2021), there have been more than 136 million reported cases of infection and more than 2.9 million deaths. However, the number of cases is underestimated, as many people do not show disease symptoms (typically, fever) upon infection and thus go undiagnosed. Whereas public attention has mostly focused on patients with severe disease, these patients only represent a small percentage of infections, and most SARS-CoV-2 infections have been asymptomatic or cases of mild disease [1]. In several community seroprevalence studies, the prevalence of antibodies to SARS-CoV-2 in healthy individuals has ranged from 2.1% to 24.4%, depending on the occupations of participants [2–6]. Therefore, it is believed that the asymptomatic infection rate could be substantially higher than documented.

It is expected that when a sufficiently large proportion of the population in a given area consists of clinically recovered COVID-19 patients, the community might become immune against SARS-CoV-2 reinfection, a phenomenon termed herd immunity. However, most studies have focused on clinical patients, and there are few reports on asymptomatic cases. Moreover, the strength of immune protection and how long it lasts in asymptomatic SARS-CoV-2 infection cases are currently unknown. These questions need to be answered to understand the possibility of herd immunity. In COVID-19 patients, increased antibody-secreting cells (ASCs), which are key for rapid antibody production following SARS-CoV-2 infection, can be observed [7]. Meanwhile, cellular responses result in the production of a large amount of cytokines, such as IL6, and a dramatic decrease in blood lymphocytes (lymphopenia) [8,9]. At the end of the disease course (normally 2 weeks), most patients recover with a subsequent decline in cellular responses but long-lasting antibody responses [8]. However, whether asymptomatic subjects share these immune features has seldom been investigated.

A recent single-center, small-scale study demonstrated a relatively weak and declining serological response in asymptomatic carriers compared to that in symptomatic COVID-19 patients but did not provide a mechanistic explanation [10]. One relevant question is whether these findings apply to all asymptomatic subjects. In the current study, we aimed to determine the proportion of past cases of asymptomatic infection in Wuhan, China, by conducting a serosurvey. We also determined viral genome sequences and characterized serological and cellular responses related to antibody production in 24 asymptomatic subjects, with the aim of better understanding the serological features of this population.

## Materials and methods

### Sample collection

Serum samples were collected from 508 healthy food workers from the Wuchang district in Wuhan in April 2020, when the outbreak was under control. Whole blood samples and sputum samples from 24 asymptomatic subjects were obtained from the Wuhan Municipal Health Commission at the late May to early June 2020, which is around 2 months after Wuhan reopen. These asymptomatic subjects were enrolled based on the following criteria: they don’t have history of hospital admission or showing any symptom of COVID-19 during the pandemic; they don’t have contact history with a confirmed case; they were detected as SARS-CoV-2 nt positive during routine body check and didn’t show symptoms of disease in the following two weeks. As controls, whole blood samples from 14 acute phase COVID-19 patients, 5 2-month recovered patients and 7 healthy donors were collected from Wuhan Pulmonary Hospital.. Serum samples from 60 symptomatic patients, who had recovered 2–3 months after initial infection and had similar disease courses as asymptomatic subjects, were also collected from Wuhan Pulmonary Hospital. Serum was separated by centrifugation at 3,000 × *g* for 15 min and inactivated at 56 °C for 1 h. The ethics committees of the designated hospitals for emerging infectious diseases approved all procedures used to obtain human samples. Written informed consent was obtained from each patient.

### Detection of SARS-CoV-2 viral RNA

RNA was extracted and tested by real-time RT–PCR with SARS-CoV-2-specific primers and probes (Vazyme). The tests were carried out in biosafety-level 2 facilities at the Wuhan Institute of Virology. A case was considered laboratory-confirmed if the sample was positive for two targets (ORF1ab or N) by real-time RT–PCR. A cycle threshold (Ct) value <40 was defined as a positive test result.

### Enzyme-linked immunosorbent assay (ELISA)

Serum IgM or IgG antibodies against the SARS-CoV-2 spike protein receptor-binding domain (RBD) or nucleocapsid protein (NP) were measured using an ELISA kit generated in-house [11,12]. Virus surrogate neutralization assays were performed using the cPASS SARS-CoV-2 Neutralization Antibody Detection Kit (catalog #L00847; GenScript) [13]. Serum cytokines were tested using commercial ELISA kits (Proteintech, Wuhan, China) according to the manufacturer’s instructions, using sera at a 1:4 dilution. OD values at 450–630 nm were determined, and cytokines were quantified based on a standard curve.

### Peripheral blood cell (PBC) preparation and flow-cytometric analysis

Blood samples from asymptomatic subjects, symptomatic cases, and healthy donors were processed in a BSL3 lab at the Wuhan Institute of Virology. The blood samples were lysed using eBioscience^TM^ 10× RBC Lysis Buffer (Multi-species), as per the general protocol. The lysis buffer was diluted 1:10 with reagent-grade water at room temperature. One milliliter of human blood sample was centrifuged at 500 × *g* at room temperature for 10 min and then added to 10 mL of the 1× RBC lysis buffer. The samples were incubated at room temperature for 10–15 min. To remove residual red blood cells, the samples were centrifuged at 500 × *g* for 10 min and then treated with another 5 mL of 1× RBC lysis buffer at room temperature for 10 min.

Antibody secreting cells (ASCs) were tested as following. The PBCs were spin-washed three times (500 × *g* for 10 min each time) with PBS containing 2% BSA before staining with cell marker antibodies. For surface staining, the PBCs were incubated with the following human-specific fluorochrome-labeled antibodies before fixing and removal from the BSL3 lab: APC/CY7-anti-CD3 (UCHT1), percp-anti-CD19 (HIB19), APC-anti-CD38 (HIT2), PE-anti-CD27 (O323), BV510-antiCD4 (OKT4), PacificBlue-anti-CD8a (RPA-T8), Percp/Cy5.5-anti-CD185 (J252D4), APC-anti-CD279 (EH12.2H7), and PE-anti-CD278 (C398.4A) at room temperature in the dark for 30 min. Antibody-stained PBCs were washed three times with PBS containing 2% BSA, fixed with 4% PFA at 4 °C overnight, and taken out of the BSL3 lab for subsequent analysis.

SARS-CoV-2 RBD specific B-lymphocyte responses were detected. In brief, thawed PBMCs (vaccinated) or PBCs (asymptomatic) were stained with human Fc blocker (Biolegend Cat#422302) diluted in PBS buffer and incubated for 10 min at room temperature. Soluble S-tagged S-RBD protein (in-house made) was then added to cells for 30 min at room temperature, and followed by incubation with anti-S-tagged antibody (in-house made) at 4 °C for 30 min. The cells were incubated with anti-mouse IgG PE conjugated antibody (Proteintech Cat#SA00003-1), live/dead staining and APC/CY7-anti-CD3 (UCHT1) and percp-anti-CD19 (HIB19) before fixation and removal from the BSL3 lab. Cells were washed after each staining. Notably, blood cells from vaccinated people were used as control, as we don’t have cells from symptomatic cases due to the successful control of COVID-19 in China.

### Full-length genome sequencing and phylogenetic analysis

Three sputum samples from three asymptomatic subjects on May 30, 2020 (AP43) or June 1, 2020 (AP44 and AP45) were collected for genome sequencing. Viral RNA genome sequences were determined by next-generation sequencing. Sequencing libraries were constructed using the ATOPlex SARS-CoV-2 Full Length Genome Panel V1.0 (MGI, BGI-Shenzhen, China), following the manufacturer’s instructions, and sequenced in an MGIseq 2000 fast sequencer. The reads were assembled into genomes using Geneious (v10.2.6) and MEGAHIT (v1.2.9). Viral clades were determined based on the GISAID database. In total, 1,927 complete SARS-CoV-2 genomes with high coverage and diverse collection dates from six different clades were randomly selected and downloaded from GISAID (https://www.gisaid.org/), and these were aligned to the three patient-derived viral genomes in this study using MAFFT (v7.407). A maximum likelihood-based phylogenetic tree was constructed in CIPRES (http://www.phylo.org/index.php) using RAxML-HPC2 based on XSEDE (v8.2.12) and was modified in FigTree (v1.4.3).

### Statistical analysis

Statistical analysis was performed using GraphPad Prism software. Data are reported as the mean ± standard deviation (SD). Means were compared using paired or unpaired two-tailed Student’s t-tests or Mann–Whitney tests. *P* < 0.05 was considered significant.

## Results

### Serological investigation of SARS-CoV-2 in food workers in wuhan

**Figure 1.**
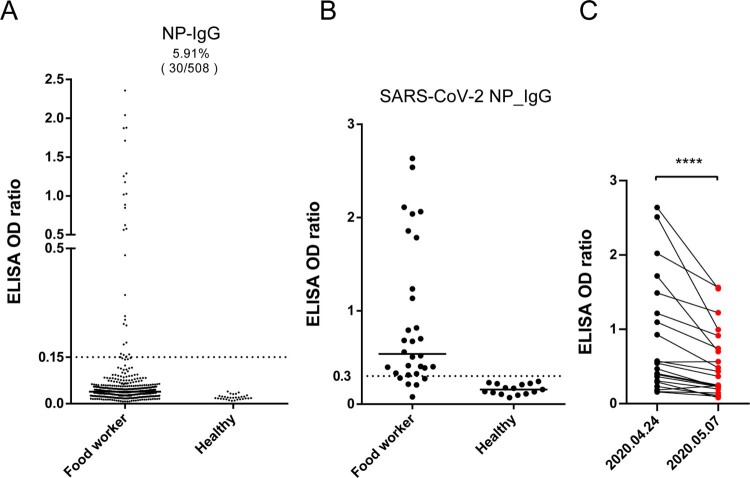
Serological investigation of SARS-CoV-2 in food workers in Wuhan. (A) Enzyme-linked immunosorbent assay (ELISA) screening of SARS-CoV-2 nucleocapsid protein (NP) IgG antibodies (n=508 subjects). The cutoff (dashed line) was five times the mean value of negative samples. Serum samples from healthy donors were used as the control (n=30). (B) Same ELISA test using a commercial kit. (C) NP IgG antibody levels in two consecutive serological tests (n=21).

Given that the COVID-19 epidemic emerged in the Hunan seafood market in Wuhan, we reasoned that food workers might be at a higher risk of SARS-CoV-2 infection than other populations. We conducted a general serological investigation of SARS-CoV-2 in food workers in a local community in Wuhan immediately when the outbreak was under control, on April 24, 2020. In total, 508 subjects who had not shown any signs of SARS-CoV-2 infection were enrolled for the detection of SARS-CoV-2 NP-specific antibodies. The data revealed that 30 of the 508 subjects (5.91%), but not healthy donors, carried viral NP antibodies in their serum, indicating that they probably had been infected with SARS-CoV-2 but were asymptomatic (Figure 1A and 1B). Notably, none of them were viral nucleotide-positive at the time of investigation.

As antibody levels in some COVID-19 patients reportedly decrease over time [14], we questioned whether such decreases would also occur in asymptomatic subjects. Therefore, we conducted a second serological test on 21 of the 30 viral NP-positive subjects 2 weeks after the first test. The data showed that the antibody levels in most of these subjects (19/21, 90.4%) had decreased by up to 50% (Figure 1C).

### Viral genomes from asymptomatic subjects

**Figure 2.**
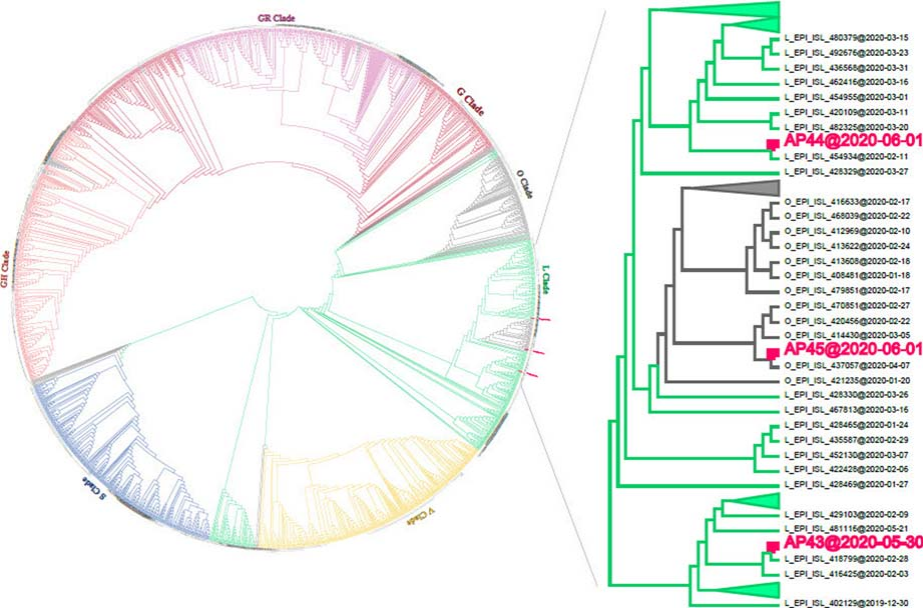
Phylogenetic tree of SARS-CoV-2 genomes. Three full-length genomes were obtained from three asymptomatic subjects collected on May 30 (AP43) or June 1 (AP44 and AP45) 2020 and were clustered with 1,927 publicly available representative genomes downloaded from the GISAID database. Sampling time is indicated at the end of each genome.

To further characterize the immune responses of asymptomatic individuals, 24 subjects admitted to a government-designated hospital who tested positive by RT–PCR in the prevalence survey, but without any relevant clinical symptoms in the preceding 14 days, were enrolled. Virus isolation was not attempted because of the low viral RNA levels in their samples (Ct >33 for all samples, Supplementary Table 1). Three full-length genomes were obtained from sputum samples of three asymptomatic subjects on May 30, 2020 (AP43) or June 1, 2020 (AP44 and AP45). Phylogenetic analysis was carried out using 1,927 SARS-CoV-2 genomes downloaded from GISAID and the three genomes obtained in this study. All three genomes clustered with early L or other related clades, and two were closely related to viral genomes sequenced in February (AP43 and AP44), whereas the third was related to a genome obtained in April, 2020 (AP45) (Figure 2). As there have been no new COVID-19 cases since April 8, 2020, the three asymptomatic subjects were probably infected between February and April and continued to shed viral RNA until May–June 2020.

### Asymptomatic subjects exhibit weak serological responses

**Figure 3.**
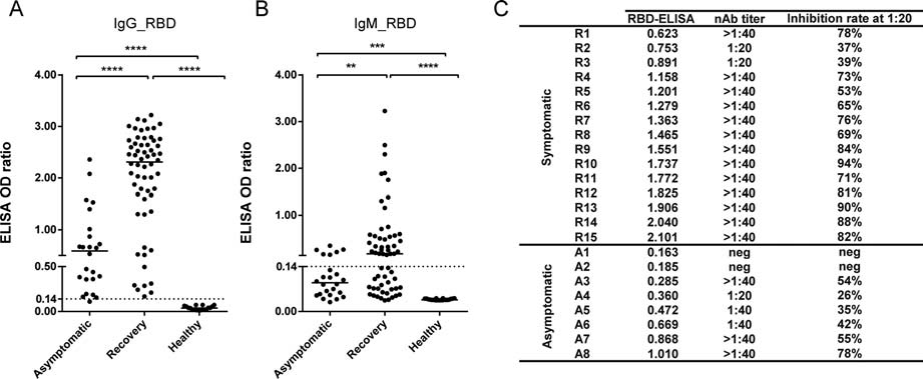
Asymptomatic subjects exhibit a weak serological response. (A, B) Comparison of IgG_RBD (A) and IgM_RBD (B) between asymptomatic subjects (n=24) and recovered symptomatic patients (n=60). Serum samples from healthy donors were used as controls (n=20). The median values are shown. Statistical significance was calculated using an unpaired Student’s *t-*test, **P < 0.01; ****P < 0.0001. (C) Neutralizing titers of selected serum samples from asymptomatic and symptomatic patients. IgG_RBD ELISA values and neutralizing antibody (nAb) titers are shown. The titer was tested using surrogate neutralization assays, shown as inhibition ratio (column C).

We compared viral-specific neutralizing or non-neutralizing antibody responses between asymptomatic and symptomatic patients who had recovered from infection. Sixty symptomatic patients who had recovered 2–3 months after initial infection and had a disease course similar to that of asymptomatic subjects were enrolled. Viral antibodies against RBD, an important viral neutralizing target, were tested. In the asymptomatic group, 95.8% (23/24) tested positive for IgG, and 100% (60/60) of the symptomatic group participants tested positive for IgG (Figure 3A). Further, 25.0% (6/24) of individuals in the asymptomatic group were positive for IgM, whereas 73.3% (44/60) of those in the symptomatic group were IgM-positive (Figure 3B). Notably, both IgM and IgG antibody levels were significantly lower in the asymptomatic group than in the symptomatic group or healthy donors (Figure 3). Similarly, the neutralizing antibody level was relatively low in the asymptomatic group (Figure 3C). It thus appears that asymptomatic subjects only generate weak serological responses against the virus, although they continue to shed viral RNA.

### Asymptomatic subjects exhibit weak ASC responses

**Figure 4.**
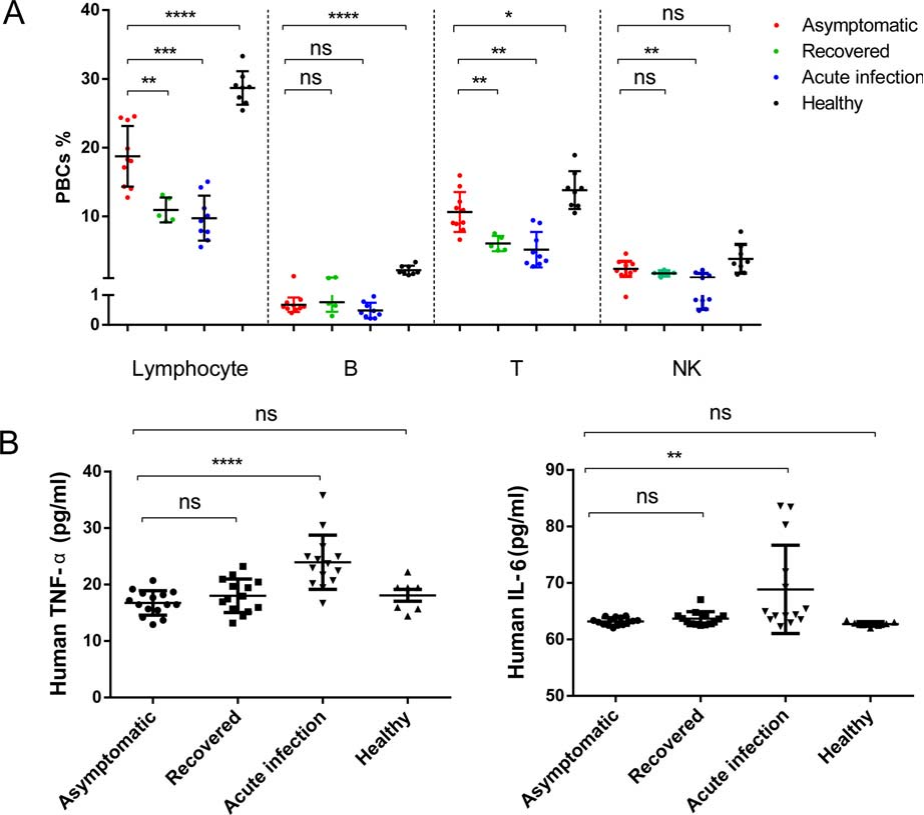
Lymphocyte ratio and signature cytokine levels in blood. (A) Flow-cytometric analysis of major lymphocyte populations in peripheral blood cells (PBCs) in asymptomatic subjects (n=10), 2-month recovered symptomatic patients (n=5), symptomatic acute infection (n=9) and healthy individuals (n=8). Data are shown as percentages of PBCs. (B) Comparison of blood IL-6 (left) and TNF-α (right) concentrations between asymptomatic subjects (n=15), 2-month recovered symptomatic patients (n=14), and symptomatic acute infection (n=14) or healthy donors (n=7). Human sera were used at 1:4 as recommended by the ELISA kit manufacturer. Mann-Whitney tests were used. Means ± SDs are shown. *P < 0.05; **P < 0.01; ***P < 0.001; ****P < 0.0001; ns, no significance.

Hallmarks of acute SARS-CoV-2 infection include lymphopenia and an increase in inflammatory cytokines, which occur in most symptomatic cases and alleviated in recovered patients. We then tested blood lymphocyte ratios (n=10) or IL6 and TNFα blood cytokine signatures (n=15) in asymptomatic subjects, in comparison to those in 2-month recovered patients, symptomatic cases and healthy donors. The results showed that all of these patients had slight lymphopenia, and their B cells were decreased more dramatically than their T or NK cells (Figure 4A). Collectively, lymphopenia or the decrease of immune cell subsets was more severe in symptomatic cases than in recovered patients, or in asymptomatic cases (Figure 4B). Consequently, IL6 and TNFα appeared normal in asymptomatic subjects and recovered patients, in contrast to the elevated responses observed in symptomatic patients (Figure 4C and 4D).

**Figure 5.**
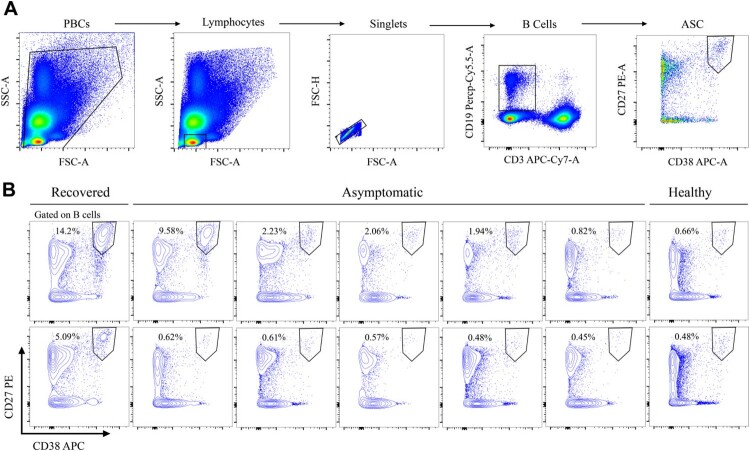
Frequency of CD3^−^CD19^+^CD27^hi^CD38^hi^ antibody-secreting cells (ASCs). (A) Gating strategy for ASCs in flow cytometry. (B) Frequency of ASCs in asymptomatic subjects (n=10), compared to that in recovered symptomatic individuals (positive control, n=2) or healthy donors (negative control, n=2).

CD3^−^CD19^+^CD27^hi^CD38^hi^ ASCs, a good indicator of antibody production capacity in a patient, are elevated following acute infection or vaccination and are prominently present during convalescence [7]. We thus tested the ASC population levels in symptomatic patients, recovered symptomatic individuals, and healthy participants (Figure 5). Whereas ASCs were prominently present in recovered symptomatic patients, most asymptomatic subjects maintained a level similar to that in healthy individuals (Figure 5B). In addition, most of the asymptomatic cases only generated minimal amount of SARS-CoV-2 RBD specific B-lymphocyte responses (Supplementary Figure 1). Together, the decrease in the B-cell ratio, low cytokine response, and low frequency of ASCs might affect antibody responses, eventually leading to the low antibody levels detected in asymptomatic subjects with SARS-CoV-2 infection.

## Discussion

We conducted a serological investigation of asymptomatic subjects with SARS-CoV-2 infection. Our results indicated a relatively high seroprevalence of asymptomatic SARS-CoV-2 infections in food workers in Wuhan, China. In addition, we found low and declining antibody responses in asymptomatic subjects, who might be vulnerable to SARS-CoV-2 reinfection when antibodies diminish. The risk of reinfection has also been demonstrated in a recent case report [15].

In several population-based studies, the seropositivity rate in healthy individuals varies, from 4.8% in Switzerland and 5.0% in Spain to 8.3% in Brazil [2,4,6]. The prevalence of asymptomatic SARS-CoV-2 infections is largely unknown in Wuhan, the original epicenter of the outbreak [16]. In a serosurvey of SARS-CoV-2 among hospital visitors in Wuhan from January to April 2020, the seroprevalence of IgG/IgM was determined to be 2.1% [3]. In comparison, the 5.91% seropositivity rate in food workers was relatively high, indicating that they have a higher probability of virus exposure than other populations, except healthcare workers. This occupational risk of infection has also been observed in a MERS-CoV serosurvey, in which the seroprevalence was 23 times higher in camel slaughterhouse workers than in other populations [17]. Likewise, several small-scale outbreaks in China originated from seafood workers who were frequently exposed to SARS-CoV-2-contaminated seafood products [18]. Notably, this level of population immunity is still substantially lower than the level needed for herd immunity.

As the strength and duration of immunity after infection are key factors for herd immunity, a weak and declining antibody response in asymptomatic subjects, as shown in our data, is important for decisions on when to ease social restrictions. Similarly, in a recent paper, the authors monitored antibody responses at 1.3 and 6.2 months after infection in a group of confirmed cases. They found that IgG and IgM responses, regardless of the existence of RBD or NP antibodies, decreased dramatically over time [19]. The antibody levels in asymptomatic subjects, specifically IgM and IgG (NP or RBD), were significantly lower than those in recovered symptomatic patients. Although we do not know the exact time of infection for the asymptomatic subjects, we assume they were most likely infected months prior, as genomes obtained from these patients in late May–June 2020 clustered with viral strains from February to April 2020. In addition, there has been no reported case of SARS-CoV-2 infection since April 8, 2020, nor there has reported symptoms of COVID-19 in these asymptomatic cases during the pandemic. Lastly, either blood lymphocytes profiles or the level of key inflammatory factors in asymptomatic cases are similar to 2-month recovered patients. Collectively, we believe these asymptomatic subjects were infected months ago based on epidemiological, virological or immunological evidences, but only developed low level of immune memory compared to recovered symptomatic patients.

Whereas we do not know the exact mechanism behind the low and declining antibody levels in asymptomatic subjects, we can speculate on some possible explanations. First, viral RNA levels were very low in these subjects, indicating that viral replication or viral-related immune responses were limited. Similarly, these subjects had undetectable levels of blood IL6 and TNFα, which are two hallmark cytokines present during symptomatic SARS-CoV-2 infection. The low levels of virus and cytokines are probably related to an asymptomatic nature of these subjects. Second, the blood B-cell ratio was decreased, which probably had a negative effect on antibody production, although the mechanism underlying this decrease is unknown. Third, asymptomatic subjects had a low ASC frequency compared to that in recovered symptomatic individuals. ASCs are normally increased with acute infection and are obviously present during convalescence [7]. However, this cell population was not prominent in most asymptomatic subjects, which probably led to low and unsustained antibody levels. As a consequence, we observed a significant difference in antibody levels between asymptomatic and symptomatic groups at 2–3 months post-infection. A similar finding has been reported by other groups [10]. It remains unknown whether SARS-CoV-2 disrupted the antibody production machinery or whether the cells had not yet been adequately activated.

In summary, we showed that asymptomatic subjects exhibit only weak and declining antibody responses against SARS-CoV-2 and thus might be vulnerable to reinfection. Neither the number of cases nor their antibody features supported herd immunity in the community of these individuals. Notably, the relatively low number of cases in this study should be considered when drawing conclusions. Viral-specific T-cell responses should also be investigated to fully understand the adaptive immune responses in asymptomatic subjects in the future. Nonetheless, social distancing and strict community measures should be continued until an effective vaccine against SARS-CoV-2 is available.

## Supplementary Material

Supplementary_Figure_1-210312.tif

Sup_table_1-Peak_viral_Ct-asymptomatic_patients.xlsx
