# Supplementary figures and images for: Serological investigation of asymptomatic cases of SARS-CoV-2 infection reveals weak and declining antibody responses
Source: Emerg Microbes Infect. 2021 May 18;10(1):905–12. doi: 10.1080/22221751.2021.1919032 (PMC8143642; doi:10.1080/22221751.2021.1919032)

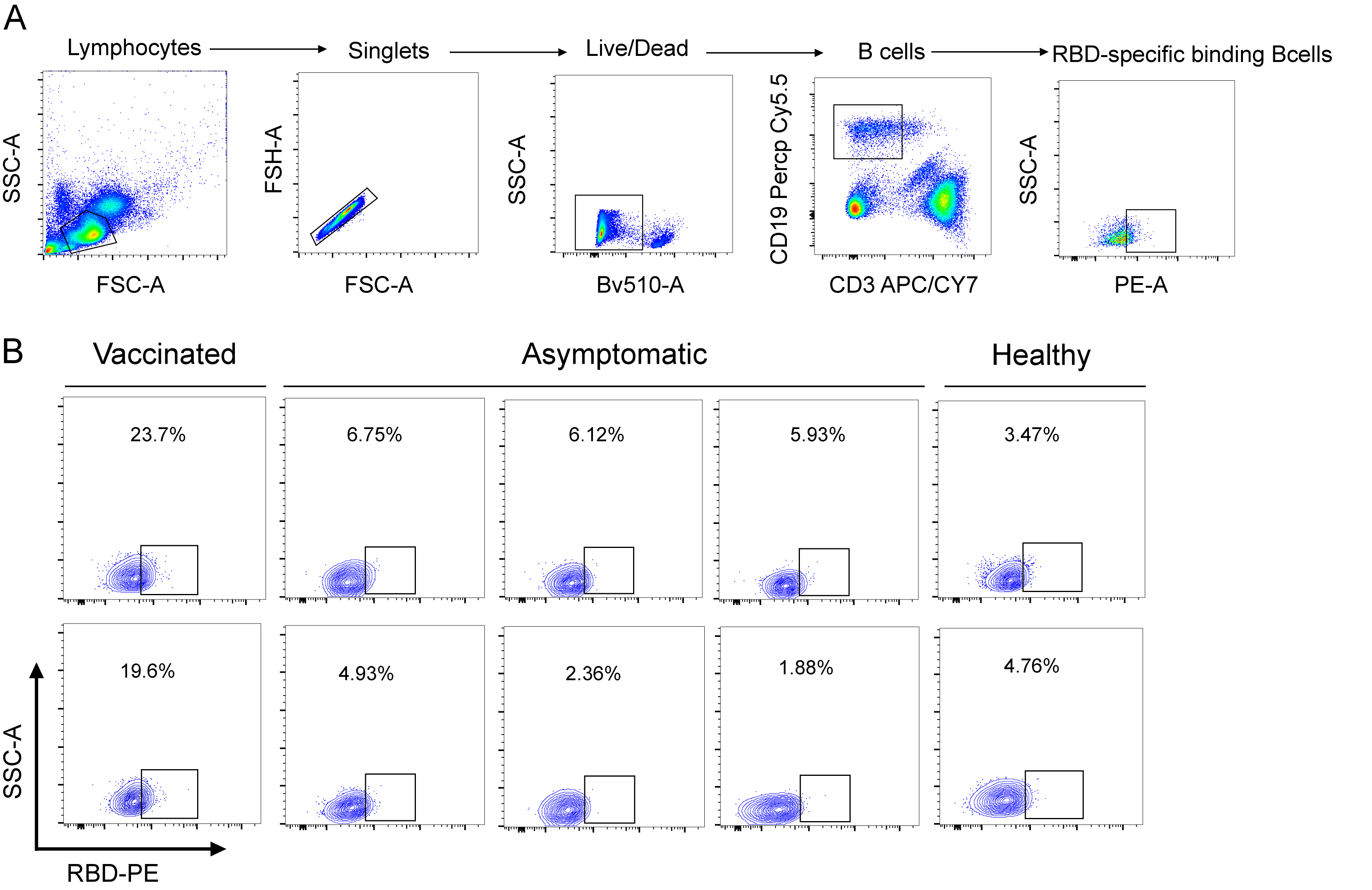

Supplement: Supplementary_Figure_1-210312.tif [file TEMI_A_1919032_SM4524.tif]
